# A case report of human immunodeficiency virus-associated anaplastic lymphoma kinase protein-negative anaplastic large cell lymphoma

**DOI:** 10.1186/2193-1801-2-400

**Published:** 2013-08-23

**Authors:** Hiroaki Taniai, Norihiro Furusyo, Masayuki Murata, Fujiko Mitsumoto, Motohiro Shimizu, Kazuhiro Toyoda, Eiichi Ogawa, Mosaburo Kainuma, Kyoko Okada, Jun Hayashi

**Affiliations:** Department of General Internal Medicine, Kyushu University Hospital, 3-1-1, Maidashi, Higashi-Ku, Fukuoka, 812-8582 Japan

**Keywords:** Human immunodeficiency virus, Anaplastic large cell lymphoma, Anaplastic lymphoma kinase

## Abstract

Human immunodeficiency virus (HIV)-associated anaplastic large cell lymphoma (ALCL) is not so common, and anaplastic lymphoma kinase protein (ALK)-negative ALCL is rare and has a low survival rate. We report a case of a 31-year-old Japanese man diagnosed with HIV-associated ALK-negative ALCL who presented with long-lasting fever of unknown origin. The diagnosis was based on a full work-up that included inguinal lymph-node biopsy. Eight-cycle chemotherapy that included cyclophosphamide, doxorubicin, vincristine, and prednisone in addition to antiretroviral therapy for HIV infection provided a complete remission of his ALCL and over 5-year survival for him.

## Introduction

It is well documented that human immunodeficiency virus (HIV)-infected patients have high rates for the development of various malignant diseases in comparison with healthy people (Sigel K et al., 2011). Acquired immunodeficiency syndrome (AIDS) patients often develop *Kaposi’s* sarcoma and non-*Hodgkin* lymphomas (NHLs). However, there are an increasing number of cases complicated with malignant diseases, such as other malignant lymphomas (ML), leukemia, and other various carcinomas even in non-AIDS patients (Lewden C et al., 2008; Philips AA et al., 2009; Simard EP et al., 2010).

Few reports of T-cell lymphoma in HIV-infected patients have been presented (Arzoo KK et al., 2004; Castillo JJ et al., 2011). Anaplastic large cell lymphoma (ALCL) is a distinct subtype of peripheral T-cell lymphoma (PTCL) characterized by the expression of CD30 in lymphoma cells. Like aggressive B-cell NHLs, the risk of developing PTCL is also increased in the setting of HIV infection. HIV-associated ALCL cells rarely expressed anaplastic lymphoma kinase protein (ALK) (Perez K et al., 2010). The 5-year overall survival of ALK- positive and -negative patients is 79% and 46%, respectively (Gascoyne RD et al. 1999). We here report a 31-year-old Japanese man with HIV infection who was diagnosed with ALK-negative ALCL with complete remission and long-time survival by antiretroviral therapy (ART) for HIV and chemotherapy.

## Case presentation

A 31-year-old Japanese man who had sex with men complained of lasting fever over 38°C, severe general fatigue, and an 8.5% reduction of body weight over the preceding four months, for which he had visited a nearby hospital in March 2008. A HIV screening test was positive and he was transferred to Kyushu University Hospital after 10 days at the original hospital. On admission, physical examination revealed a fever of 39.8°C, blood pressure 110/69 mmHg, pulse rate 110 beats per min, body weight 54 kg, height 168 cm, and body mass index 19.1 kg/m^2^. Examination of the oral cavity showed small mucosal nodules, suggesting oral *Candidiasis* and mild hepatosplenomegaly without any other lymphadenopathy.

Table 1 shows the patient’s biochemical laboratory data on admission. The CD4 cell count and HIV viral load were 26/μL and 100,000 copies/mL, respectively. *Epstein-barr virus* and *Cytomegalovirus* infection were ruled out in his sera. Plasma β-D glucan and other antigens for fungal infections, such as *Cryptococcus neoformans* and *Toxoplasma gondii,* were negative, and cultures taken several times from blood, urine, and sputum were all negative.

**Table 1:** **Laboratory data on admission**

Red cell count (/mm^3^)	358 × 10^4^	Rapid plasma reagin	Negative
Hemoglobin (g/L)	93	TPHA (titer)	320
White-cell count (/mm^3^)	2680	*Chlamydophila trachomatis* IgG/IgA	Positive / Negative
Platelet count (/mm^3^)	4.9 × 10^4^	Soluble interleukin II receptor (U/mL)	2056
Erythrocyte sedimentation rate (mm/hr)	81	*Cryptococcus* Antigen (U/mL)	<3
Serum Creatinine (mg/L)	6.7	*Toxoplasma* Antibody IgG (U/mL)	<100
Serum glucose (mg/dL)	101	*Aspergillum* Antibody IgG (U/mL)	<0.1
Serum total bilirubin (mg/L)	4.0	Quanti FERON-E (U/mL)	0.12
Serum total protein (g/L)	74	Quanti FERON-C (U/mL)	0.1
Serum albumin (g/L)	31	β-D glucan (pg/mL)	4.01
Alkaline phosphatase (U/L)	246	EBV Early antigen IgG	Negative
Aspartate aminotransferase (U/L)	51	EBV Capsid antigen	Negative
Alanine aminotransferase (U/L)	43	EBV Nuclear antigen -IgG	Negative
Lactate dehydrogenase (U/L)	432	CMV Antibody IgG	80
Serum amylase (U/L)	127	CMV Antibody IgM	<0.1
Serum creatine kinase (U/L)	99	CMV antigenemia (C7HRP)	Negative
Serum CRP (mg/dL)	2.94	HIV RNA (copies/mL)	100,000
HBs antigen	Negative	CD4 (/μL)	26
HCV Antibody	Negative	CD8 (/μL)	132
HTLV-1 Antibody	Negative	CD4/CD8 ratio	0.2

*HBs* hepatitis B surface, *HCV* Hepatitis C virus, *HTLV* Human T cell leukemia virus, *TPHA Treponema pallidum* hemaggulutination assay, *EBV Epstein-Barr* virus, *CMV Cytomegalovirus*.

Imaging tests such as chest and abdominal X-ray; cervical, thoracic and abdominal computed tomography (CT) scans; head, trunk, and total spinal magnetic resonance imaging (MRI); and gallium scintigraphy were done. Thoracic CT showed some small masses (<5 mm) in the bilateral lower lobes of the lung. The first MRI and gallium scintigraphy showed high signals in the spine and sacroiliac joint, but indicated no other specific findings. Bone marrow aspiration showed normo to hypercellular marrow tissue. A lower gastrointestinal endoscopy showed no other abnormalities, such as malignant diseases.

Although there was no evidence of *Mycobacterium avium* complex (MAC) infection, we suspected MAC infection and at day 2 after admission and began an oral, multi-drug regimen that included rifampicin 600 mg/day, clarithromycin 800 mg/day, and ethambutol 750 mg/day. Because these treatments were not effective, we considered the possibility of HIV-associated fever, and started ART with raltegravir, tenofovir/emtricitabine, and lopinavir/ritonavir at hospital day 13 after admission. After induction of ART therapy, body temperature once decreased to under 37°C, but again increased to over 39°C after a few days. At 12 days after induction of ART, his CD4 cell count increased from 26/μL to 72/μL and HIV viral load decreased from 100,000 copies/mL to 59 copies/mL. Considering the possibility of drug induced fever, we stopped all drugs for the MAC and *Candida* infections, but fever over 39°C continued.

**Figure 1:**
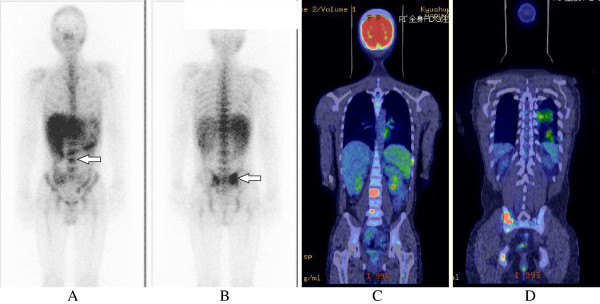
**Gallium scintigraphy at hospital day 38 and fluoro-2-deoxy-D-glucose positron emission tomography at hospital day 56.** Images of gallium scintigraphy show high up-take (arrows) in the systemic bone marrow **(1A)** and right sacroiliac joint **(1B)**. Images of fluoro-2-deoxy-D-glucose positron emission tomography **(1C and 1D)** show abnormal uptake in some ribs, the spine, sacroiliac joint, mediastinal lymph nodes, and bilateral hilum of lung.

A second thoracic CT showed another lesion in the left lung, and on that occasion his blood was positive by Quanti FERON-TB Gold In Tube assay, which is used for the screening of latent tuberculosis. To rule out pulmonary tuberculosis, bronchoscopy was done, but the bronchoalveolar lavage indicated no pulmonary diseases. Repeated cultures of sputum, gastric fluid, and bone marrow tissue were negative for *Mycobacterium tuberculosis* and MAC. The second trunk MRI at hospital day 31 showed high signals in the spine and sacroiliac joint. At hospital day 38, the second gallium scintigraphy showed high up-take of systematic bone marrow and in the right sacroiliac joint (Figure 1A and 1B).

**Figure 2:**
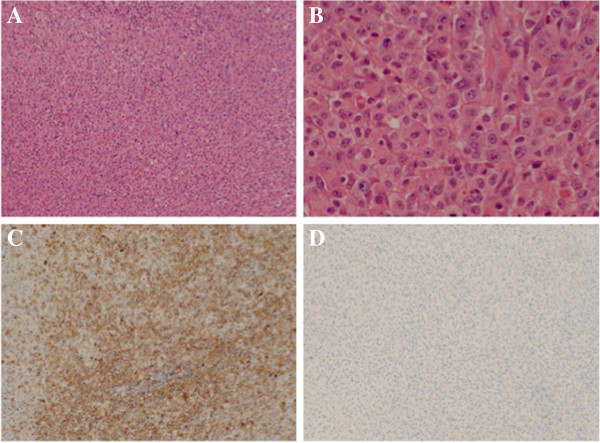
**Pathology of the right inguinal lymph node.** Panels **2A** and **2B** show hematoxylin eosin stain of the inguinal lymph node. Panel **2C** shows the stain for CD 30 and Panel **2D** for anaplastic lymphoma kinase (ALK).

At hospital day 56, fluoro-2-deoxy-D-glucose positron emission tomography revealed abnormal uptake in some ribs, the spine, sacroiliac joint, mediastinal lymph nodes, and bilateral hilum of the lung (Figure 1C and 1D). We strongly suspected malignant lymphoma, although there was little up-take in the inguinal lymph node. Because of newly palpable swelling in the right inguinal lymph node, biopsy was done. The tissue section showed a variable proportion of large hallmark cells with eccentric horseshoe or kidney-shaped nuclei with an eosinophilic region near the nucleus and many scattered, atypical large lymphoid cells together with small lymphocytes (Figure 2A). Atypical lymphoid cells showed large, atypical lymphoid cells with bizarre nuclei (Figure 2B). Immunohistochemically, these cells were positive for T-cell markers (CD3, CD45RO), CD30 and CD4, but negative for B-cell markers (CD20, CD79a) and anaplastic lymphoma kinase (ALK) (Figure 2C and 2D). Other types of T-cell lymphoma, such as ATL, were ruled out. These features confirmed T-cell lymphoma of the ALK-negative ALCL type. At the same time, flow cytometry indicated the likely pattern of ALCL. The second bone marrow aspiration showed normal to hypercellular marrow tissue. The final diagnosis was HIV-associated ALK-negative ALCL (stage IV) in May 2008. His performance status was 0 and age adjusted International Prognostic Index was 3. Immediately, chemotherapy (cyclophosphamide, doxorubicin, vincristine and prednisone; CHOP) was administered in eight courses done at an interval of three weeks. There was a little reduction of the white blood cell count due to chemotherapy and no other toxicity related to the therapy was shown, thus no dose reduction was necessary at any time during the therapy. After induction of an eight-cycle course of CHOP, systemic lymph node swelling was markedly decreased and clinical symptoms and laboratory data showed complete remission. After completion of CHOP therapy, ART was continued and led to a favorable increase of CD4 cells. The patient has had no recurrence of ALCL until August 2013, suggesting the overall survival period of the patients after diagnose is 63 months.

## Discussion

The definition of ALCL has evolved since its original description in 1985 (Stein H et al., 1985), and ALCL represents a well-characterized group of T-cell lymphomas. Currently, the World Health Organization classification for ALCL represents a unique diagnostic subcategory, which comprises approximately 3% of adult and 10%-30% of childhood NHLs (Azoo KK et al., 2004). The defining features of ALCL include proliferation of predominantly large lymphoid cells with a characteristic growth pattern and strong expression of CD30. There are three groups of ALCL according to molecular and clinical criteria: Primary systemic ALK-positive anaplastic lymphoma, ALK-negative primary systemic anaplastic lymphoma, and primary cutaneous anaplastic lymphoma. ALK expression is caused by chromosomal translocation, most commonly t (2; 5) (Philips AA et al., 2009; Simard EP et al., 2010). Most ALCLs in children and younger adults express ALK protein and show favorable prognosis, while ALK-negative ALCLs are more heterogeneous and have a poor prognosis (Stein H et al., 2000). ALK-negative ALCL is highly associated with older men and shows poor prognosis (<32% 5-year survival) (Vose J et al., 2008). In the literature, HIV-associated ALCL cells rarely expressed anaplastic lymphoma kinase (Perez K et al., 2010). Moreover, the treatment response, prognosis, and long-term survival of ALK-negative ALCL are far worse than for ALK-positive ALCL (Stein H et al., 2000). For our patient, ART and CHOP were highly effective and resulted in complete remission. Some cases of HIV-associated ALCL treatment with etoposide, vincristine, doxorubicin, cyclophosphamide, and prednisone chemotherapy have been reported (Nagajothi N et al., 2007). It is unclear which chemotherapy for HIV-associated ALK-negative ALCL is the most effective due to the small number of cases. More data is needed to improve the treatment options for these patients.

There are several reports that ART has a supportive effect on chemotherapy for HIV-associated ML. Many prior studies have focused on changes in risk for more common non-AIDS-defining malignancies in HIV-infected patients undergoing ART, and more recent studies have evaluated malignancy risk with respect to individual ART use, including the effect of specific antiretroviral agents, ART drug classes, and the duration of ART. Other studies have evaluated the association of immune function and the risk associated with non-AIDS-defining malignancies, which may mediate the observed relationships of cancer risk and ART use (Silverberg MJ et al., 2009). The number of patients with HIV-associated T-cell lymphoma remains small. Therefore, better and more effective treatment options for patients with HIV-associated ALK-negative ALCL will be a welcome addition to the presently used regimens.

To date, the occurrence of ALCL in HIV-positive individuals is limited to a few case reports and small case series (Perez K et al., 2010; Genet P. 2012). A total of 37 cases of HIV-associated ALCL were identified after reviewing the available published literature (Perez K et al., 2010). Analysis of these cases showed that this group of HIV-infected patients was on average 38 years of age with a male-to-female ratio of 4:1, and a reported median CD4 cell count of 83 cells/mm^3^. *Epstein-Barr* virus infection was associated with one-third of the cases. These lymphomas manifested almost exclusively with extranodal involvement and exhibited a very aggressive clinical course. The median overall survival was only 5 months. The administration of chemotherapy and early stages at presentation were identified as good prognostic factors, while the use of ART showed a statistical trend toward improved survival in HIV-associated ALCL. Our case has suffered from stage IV ALCL with an extremely low baseline CD4 count (26/μL) not *Epstein-Barr* virus infection, but has survived more 5 years without remission by chemotherapy and ART.

## Consent

Written informed consent was obtained from the patient for the publication of this report and any accompanying images.
